# The impact of gut microbiome modulation on anthropometric indices in metabolic syndrome: an umbrella review

**DOI:** 10.1097/MS9.0000000000003140

**Published:** 2025-03-07

**Authors:** Maryam Sadat Aleali, Abinash Mahapatro, Gautam Maddineni, Ruchir Paladiya, Herby Jeanty, Elan Mohanty, Mohit Mirchandani, Ali Jahanshahi, Pavan Devulapally, Azin Alizadehasl, Muhammad Daoud Tariq, Seyedeh Fatemeh Hosseini Jebelli, Azam Yalameh Aliabadi, Seyyed Mohammad Hashemi, Ehsan Amini-Salehi

**Affiliations:** a Gastrointestinal and Liver Diseases Research Center, Guilan University of Medical Sciences, Rasht, Iran; b Hi-Tech Medical College and Hospital, Rourkela, Odisha, India; c Florida State University, Tallahassee, Florida, USA; d University of Connecticut Health Center, Farmington, Connecticut, USA; e The Brooklyn Hospital Center, Brooklyn, New York, USA; f Gautam Maddineni, MD Mary Medical Center Apple Valley, Apple Valley, California, USA; g Montefiore Medical Center Wakefield Campus, Bronx, New York, USA; h Social Determinants of Health Research Center, Guilan University of Medical Sciences, Rasht, Iran; i Faculty of Medicine, Guilan University of Medical Sciences, Rasht, Iran; j Main Methodist Hospital, San Antonio, Texas, USA; k Rajaie Cardiovascular Medical and Research Center, Iran University of Medical Science, Tehran, Iran; l Department of Internal Medicine, Foundation University Medical College, Islamabad, Pakistan; m Rajaie Cardiovascular Medical and Research Center, Iran University of Medical Science, Tehran, Iran; n Cardiovascular Research Center, Faculty of Medicine, Hormozgan University of Medical Sciences, Bandar Abbas, Iran

**Keywords:** anthropometric indices, gut microbiome, meta-analysis, metabolic syndrome, prebiotics, probiotics, synbiotics, umbrella review

## Abstract

**Background:**

Metabolic syndrome (MetS) is a complex disorder characterized by a cluster of metabolic risk factors. Recent research highlights the gut microbiome’s role in metabolic regulation, suggesting that modulation through probiotics, prebiotics, and synbiotics may provide a novel approach to managing MetS. This umbrella review aims to integrate insights from existing meta-analyses to explore how changes in gut microbiota influence key body measurement indicators in individuals with MetS.

**Methods:**

A systematic search of PubMed, Scopus, and Web of Science databases identified meta-analyses that assessed the impact of probiotics, prebiotics, or synbiotics on anthropometric indices in MetS patients.

**Results:**

The results indicated that microbial therapy leads to a significant reduction in body mass index (BMI) (SMD: −0.22; 95% CI: −0.35 to −0.09; *P* < 0.01) and waist circumference (WC) (SMD: −0.47; 95% CI: −0.80 to −0.15; *P* < 0.01). However, microbial therapy did not significantly affect body fat mass (SMD: −0.30; 95% CI: −0.64 to 0.02; *P* = 0.06), body fat percentage (SMD: −0.29; 95% CI: −0.62 to 0.03; *P* = 0.07), waist-to-hip ratio (SMD: −0.09; 95% CI: −0.46 to 0.28; *P* = 0.63), and weight (SMD: −0.06; 95% CI: −0.21 to 0.08; *P* = 0.37).

**Conclusions:**

Gut microbial modulation, mainly through probiotics and synbiotics, shows promise in reducing BMI and WC in MetS patients. However, its effects on other anthropometric indices remain uncertain, warranting further high-quality research to fully understand microbial interventions’ therapeutic potential.

## Introduction

Metabolic syndrome (MetS) is a prevalent and complex disorder characterized by a cluster of interrelated metabolic risk factors, including central obesity, dyslipidemia, hypertension, and insulin resistance^[1-4]^. This syndrome significantly elevates the risk of developing cardiovascular diseases, type 2 diabetes, and other serious health conditions^[5-10]^. The increasing global prevalence of MetS and other metabolic diseases poses a substantial public health challenge, necessitating effective interventions to manage and mitigate its associated risks^[6,11-15]^.

**HIGHLIGHTS**

- Metabolic syndrome (MetS) significantly increases the risk of cardiovascular diseases and type 2 diabetes, posing a major public health challenge.
- Our umbrella review demonstrates that gut microbial modulation, particularly with probiotics and synbiotics, significantly reduces BMI and waist circumference in individuals with MetS.
- Integrating probiotics and synbiotics into MetS management strategies may offer effective adjunctive therapy, but further research is necessary to confirm benefits across other anthropometric indices.

Recent advances in understanding the gut microbiome’s role in human health have unveiled its potential influence on metabolic and endocrine functions^[16-20]^. The gut microbiome, a complex community of microorganisms residing in the gastrointestinal tract, is integral to numerous physiological processes, including digestion, immune function, and energy homeostasis^[21-24]^. Dysbiosis, or the imbalance in the gut microbial community, has been implicated in the pathogenesis of various metabolic disorders, including obesity, type 2 diabetes, and, more recently, MetS^[25-27]^.

Emerging evidence suggests that gut microbial modulation through probiotics, prebiotics, synbiotics, and dietary modifications may offer a novel approach to managing MetS and its associated metabolic disturbances^[28-30]^. These interventions aim to restore a healthy gut microbiota composition, thereby improving insulin sensitivity, reducing inflammation, and potentially influencing body weight and other anthropometric indices^[31-33]^. Given the complex nature of MetS and its associated metabolic issues, it is essential to understand how gut microbiome modulation affects various anthropometric indices, including body mass index (BMI), waist circumference (WC), hip circumference (HC), waist-to-hip ratio (WHR), body fat percentage (BFP), and body fat mass (BFM).

Although several meta-analyses have evaluated the impact of probiotics, prebiotics, and synbiotics on anthropometric indices in individuals with MetS, significant gaps remain. These include variability in study methodologies, inconsistent intervention durations, and differences in microbial strains used. Moreover, prior meta-analyses have often focused on isolated aspects of microbial interventions, such as specific probiotics, without considering the broader spectrum of microbial therapies^[28,34-36]^. An umbrella review synthesizes data from multiple meta-analyses and systematic reviews, providing more evidence and a broader perspective. This approach allows for critically evaluating the methodologies and findings across studies, highlighting areas of consensus and discrepancies and offering more robust conclusions for clinical practice^[37-41]^.

This umbrella review aims to systematically synthesize the evidence from existing studies on the effects of gut microbial modulation on anthropometric indices in patients with MetS. By collating and critically appraising the findings from diverse research designs and methodologies, this review seeks to provide a comprehensive overview of the current knowledge in this field, identify gaps in the literature, and suggest potential directions for future research.

## Methodology

### Methods

In our umbrella review, which systematically synthesizes findings from multiple meta-analyses, we adhered to the guidelines outlined in the Cochrane Handbook for Systematic Reviews^[42]^. The results were reported following the Preferred Reporting Items for Systematic Reviews and Meta-Analyses (PRISMA; Supplementary Digital Content, http://links.lww.com/MS9/A755) guidelines, ensuring clarity and rigor in presenting our findings^[43]^. The protocol of the study was registered in PROSPERO (CRD42024578855).

### Search strategy

Two independent researchers devised a thorough search strategy to identify meta-analyses on the impact of gut microbiota on anthropometric indices of patients with MetS. A comprehensive search of databases (PubMed, Scopus, and Web of Science) was conducted from their inception until 19 May 2024. Keywords included “Metabolic Syndrome,” “Insulin Resistance Syndrome X,” “Dysmetabolic Syndrome X,” “Metabolic Cardiovascular Syndrome,” “Probiotics,” “Prebiotics,” “Synbiotics,” “Meta-Analysis,” and “Systematic Review.” Two information specialists reviewed the search strategy to ensure accuracy, and references from relevant studies were manually checked. No language restrictions were applied. Any disagreements were resolved by consulting a third researcher. Study management and organization were streamlined using EndNote X20.

### Study selection and eligibility criteria

Two independent researchers conducted the study screening and selection process, and any differences in their evaluations were addressed and resolved through discussions with a third researcher. The following requirements must be fulfilled by the meta-analysis studies that were part of the study: (1) Patients with MetS made up the study’s population. (2) Probiotics, prebiotics, or synbiotics were used as part of the intervention. (3) At least one anthropometric index was measured. Reports, editorials, original research, and commentary were among the studies taken from the analysis.

### Quality assessment

We used the AMSTAR2 checklist to assess the methodological quality of the included meta-analyses. Two reviewers independently evaluated the 16 items on the checklist, marking each as “yes,” “no,” or “partial yes”^[44]^. Any disagreements were resolved by consulting a third researcher.

### Data extraction

The data extracted from the meta-analyses encompassed details such as the first author’s name, year of publication, journal, country of origin, the number of original studies included, total sample size, and the quality assessments of these studies. This information was organized in an Excel spreadsheet. To fill any gaps, corresponding and primary authors were contacted. Two researchers collaboratively handled the data extraction, with any disagreements resolved through consultation with a third researcher.

### Statistical analyses

Comprehensive Meta-Analyses software version 4 and STATA version 18 were used for the analysis. The analysis used the findings of the original studies included in each meta-analysis. Original studies were identified from each meta-analysis when there were several available meta-analyses for a given outcome. Once duplicates were removed, the remaining original study results were used to conduct our meta-analysis. Standardized mean difference (SMD) was chosen for continuous outcomes due to its ability to standardize effect sizes (ESs) across studies with heterogeneous measurement scales, facilitating meaningful comparisons between diverse studies. This approach is particularly advantageous in umbrella reviews, synthesizing findings from meta-analyses, often using varying metrics to measure similar outcomes. Additionally, SMD offers a standardized metric commonly used in meta-analytic procedures, guaranteeing methodological validity and improving the interpretability of results. Additionally, SMD facilitates advanced statistical analyses, including assessments of heterogeneity and publication bias, which are critical for evaluating the validity of meta-analytic results. Tools such as funnel plots rely on SMD to identify potential biases and assess the robustness of conclusions.

Cochrane’s *Q* test and *I*^2^ statistics were utilized to measure heterogeneity across trials, with *I*^2^ values over 50% and *P*-values under 0.1 indicating substantial heterogeneity. Publication bias was assessed by visually inspecting the funnel plot and performing Egger’s and Begg’s regression tests, both at a significance level of *P* < 0.1. Additionally, “Trim and Fill” analysis was employed to detect potential funnel plot asymmetry and ensure the robustness of the results^[45,46]^.

### Result

**Figure 1.**
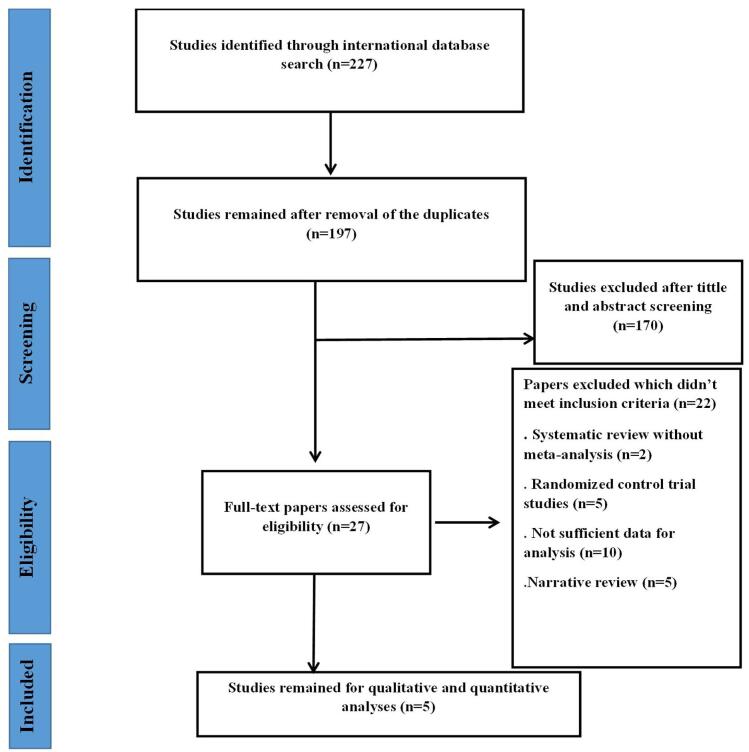
Study selection process.

An initial search of electronic databases yielded 227 studies. After removing 30 duplicates, 197 studies remained. These were screened based on their titles and abstracts, leading to the exclusion of 170 studies. Of the 22 studies that underwent full-text evaluation, only 5 met the inclusion criteria and were included in the final analysis. The selection process is depicted in Fig. 1.

### Studies characteristics

**Figure 2.**
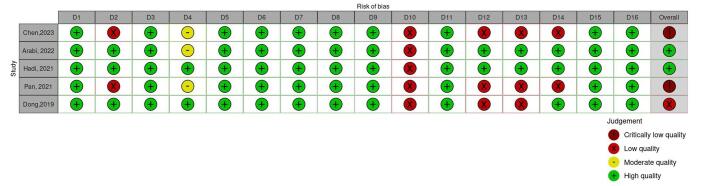
Quality of included studies.

Among the five articles analyzed, one study used probiotics as the sole intervention, while another employed synbiotics. Two studies used probiotics and synbiotics simultaneously, and one incorporated a combination of probiotics, prebiotics, and synbiotics. Two of these studies disclosed that they received funding for their research, whereas the remaining three did not report any financial support. Regarding protocol registration, three articles had pre-registered protocols with PROSPERO, while the other two studies did not have any registered protocols. The number of studies included in the meta-analyses varied significantly, ranging from 5 to 42, with total sample sizes spanning from 344 to 5,846 participants. All studies utilized the Cochrane Risk of Bias tool to assess the quality of the original studies included in their analyses. The anthropometric indices evaluated across these studies included BMI, WC, body weight, HC, WHR, BFM, and BFP (Table 1). The AMSTAR 2 assessment rated two studies as critically low quality, one as low quality, and two as high quality (Fig. 2).

**Table 1:** Characteristics of included studies

First Author name, Year of publication	Journal	Intervention	Searched Databases	Outcomes	Date of search	Number of included studies	Total sample size	The checklist used for quality assessment of included original studies	Funding status	Previously registered protocol	Model of analysis	AMSTAR 2 score
Chen, 2023^[47]^	Frontiers in Endocrinology	Probiotics and synbiotics	Web of Science, PubMed, Embase, and Cochrane Library	Body mass index	From inception up to July 2023	11	608	Cochrane Risk of Bias tool	No	No	Random effect model for heterogeneous result and fixed effect model for homogeneous result	Critically low quality
Arabi, 2022^[48]^	Pharmacological Research	synbiotics	PubMed, Scopus, and Web of Science	Waist circumference, body weight, and body mass index	From inception up to October 2021	5	1049	Cochrane Risk of Bias tool	No	PROSPERO (CRD42021276776)	Random effects model	High quality
Hadi, 2021^[49]^	Clinical Nutrition	Probiotics and synbiotics	Web of Science, PubMed, Embase, Cochrane Library	Waist circumference, body weight, and body mass index	From inception up to March 2020	10	344	Cochrane Risk of Bias tool	No	PROSPERO (RD42018116033)	Random effects model	High quality
Pan, 2021^[36]^	Frontiers in Nutrition	Probiotics, prebiotics, and synbiotics	PubMed, Cochrane Library, and Embase	Body mass index	From inception up to May 2021	42	5846	Cochrane Risk of Bias tool	Yes	No	Random effects model	Critically low quality
Dong, 2019^[34]^	Annals of Nutrition and Metabolism	Probiotics	PubMed, Cochrane Library, and CINAHL	Body mass index, waist circumference, hip circumference, waist-to-hip ratio, body fat mass, and body fat percentage	From inception up to Jan 2018	18	1544	Cochrane Risk of Bias tool	Yes	PROSPERO (CRD42018086937)	Random effect model for heterogeneous result and fixed effect model for homogeneous result	Low quality

## The results of the meta-analysis

### The effects of microbial therapy on BMI

**Figure 3.**
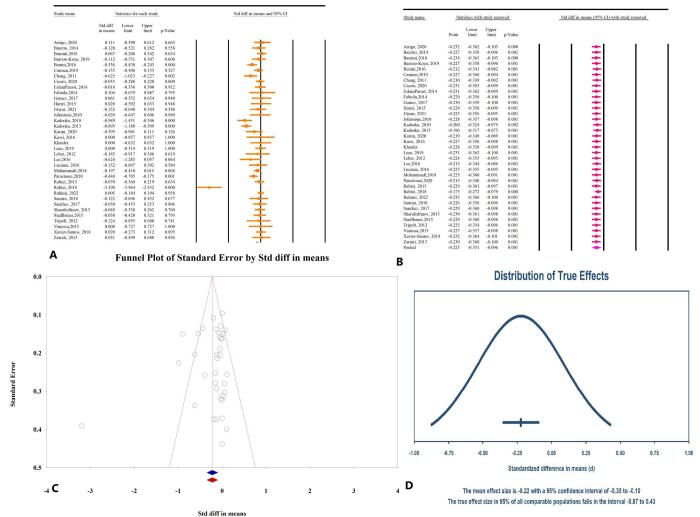
The effects of microbial therapy on BMI in patients with metabolic syndrome. A: Forest plot illustrating the overall effect of microbial therapy on BMI. B: Sensitivity analysis showing the impact of removing individual studies on the overall effect. C: Assessment of publication bias. D: Prediction interval, displaying the expected range of true effects based on the meta-analysis.

The meta-analysis results demonstrated that microbial therapy significantly reduces BMI in patients with MetS (SMD: −0.22; 95% CI: −0.35, −0.09, *P* < 0.01, *I*^2^ = 73.19%) (Fig. 3A). Subgroup analyses revealed that prebiotics (SMD: −0.03; 95% CI: −0.27, 0.19, *P* = 0.75, *I*^2^ = 0.00%), probiotics (SMD: −0.23; 95% CI: −0.37, −0.08, *P* <0.01, *I*^2^ = 60.32%), and synbiotics (SMD: −0.312; 95% CI: −0.609, −0.014, *P* = 0.04, *I*^2^ = 87.66%) can significantly reduce BMI. Sensitivity analysis indicated that the overall effect remained consistent after the removal of individual studies (Fig. 3B). Publication bias tests revealed no significant risk, with Egger’s regression test (*P* = 0.32) and Begg’s test (*P* = 0.08). Additionally, the trim-and-fill analysis, conducted without imputing any studies, was consistent with our results (SMD: −0.22; 95% CI: −0.35, −0.09) (Fig. 3C). The prediction interval analysis confirmed that the distribution of true effects ranges from −0.87 to 0.43 (Fig. 3D).

### The effects of microbial therapy on WC

**Figure 4.**
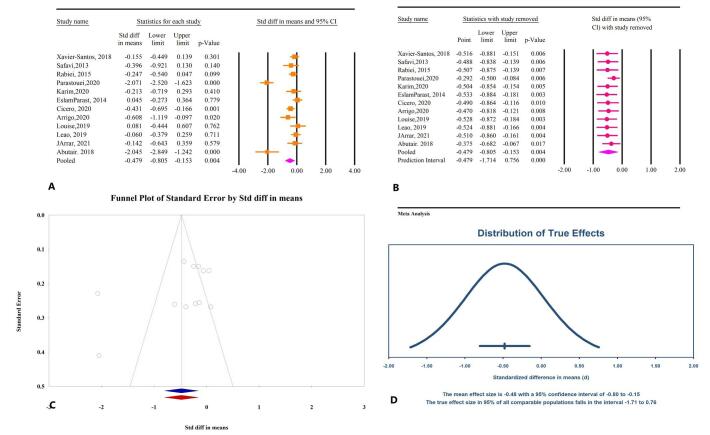
The effects of microbial therapy on WC in patients with metabolic syndrome. A: Forest plot illustrating the overall effect of microbial therapy on BMI. B: Sensitivity analysis showing the impact of removing individual studies on the overall effect. C: Assessment of publication bias. D: Prediction interval, displaying the expected range of true effects based on the meta-analysis.

The meta-analysis findings revealed a significant reduction in WC with microbial therapy in patients with MetS (SMD: −0.47; 95% CI: −0.80, −0.15, *P* < 0.01, *I*^2^ = 87.74%) (Fig. 4A). Sensitivity analysis indicated that the overall effect remained consistent after the removal of individual studies (Fig. 4B). Subgroup analysis suggested that this reduction was significant with synbiotics (SMD: −0.49; 95% CI: −0.89, −0.10, *P* = 0.01, *I*^2^ = 89.35%), while prebiotics (SMD: −0.46; 95% CI: −1.14, 0.21, *P* = 0.18, *I*^2^ = 86.43%) did not demonstrate a significant effect. Probiotics lacked sufficient research to draw definitive conclusions. Publication bias tests were insignificant, as shown by Egger’s regression test (*P* = 0.18) and Begg’s (*P* = 0.37). The trim-and-fill analysis, conducted without imputed studies, yielded an SMD of −0.47 (95% CI: −0.80, −0.15) (Fig. 4C). The prediction interval analysis further confirmed that the distribution of true effects ranges from −1.71 to 0.76 (Fig. 4D).

### The effects of microbial therapy on BFM

**Figure 5.**
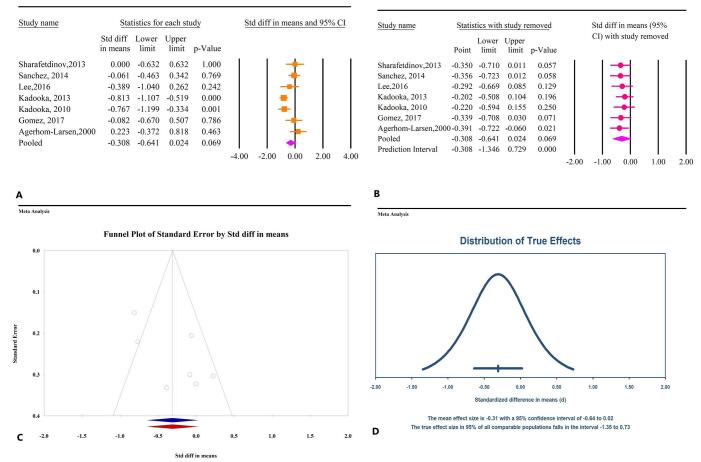
The effects of microbial therapy on BFM in patients with metabolic syndrome. A: Forest plot illustrating the overall effect of microbial therapy on BMI. B: Sensitivity analysis showing the impact of removing individual studies on the overall effect. C: Assessment of publication bias. D: Prediction interval, displaying the expected range of true effects based on the meta-analysis.

The meta-analysis results indicated that microbial therapy did not have a significant effect on BFM in patients with MetS (SMD: −0.30; 95% CI: −0.64, 0.02, *P* = 0.06, *I*^2^ = 69.73%) (Fig. 5A). Sensitivity analysis confirmed that the overall effect remained consistent even after the removal of individual studies (Fig. 5B). Notably, all studies assessing the impact of microbial therapy on BFM involved probiotics, with no studies evaluating prebiotics or synbiotics as interventions. Publication bias tests did not reveal significant bias, including Egger’s regression test (*P* = 0.32) and Begg’s correlation test (*P* = 0.08). The trim-and-fill analysis, conducted without imputing additional studies, yielded an SMD of −0.30 (95% CI: −0.64, 0.02) (Fig. 5C). The prediction interval analysis suggested a dispersion among true effects, ranging from −1.35 to 0.73 (Fig. 5D).

### The effects of microbial therapy on BFP

**Figure 6.**
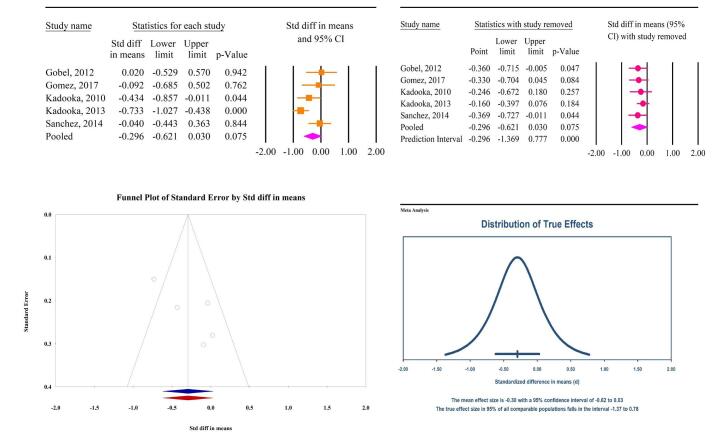
The effects of microbial therapy on BFP in patients with metabolic syndrome. A: Forest plot illustrating the overall effect of microbial therapy on BMI. B: Sensitivity analysis showing the impact of removing individual studies on the overall effect. C: Assessment of publication bias. D: Prediction interval, displaying the expected range of true effects based on the meta-analysis.

The meta-analysis findings revealed no significant effects of microbial therapy on reducing BFP in patients with MetS (SMD: −0.29; 95% CI: −0.62, 0.03, *P* = 0.07, *I*^2^ = 64.44%) (Fig. 6A). Sensitivity analysis confirmed that the overall effect remained consistent even after the removal of individual studies (Fig. 6B). Notably, all studies assessing the impact of microbial therapy on BFP involved probiotics, with no studies evaluating prebiotics or synbiotics as interventions. Publication bias tests, including Egger’s regression test (*P* = 0.08) and Begg’s test (*P* = 0.80), did not reveal significant bias. The trim-and-fill analysis, conducted without the insertion of additional studies, yielded an SMD of −0.29 (95% CI: −0.62, 0.03) (Fig. 6C). The prediction interval analysis indicated a dispersion among true effects, with prediction intervals ranging from −1.37 to 0.78 (Fig. 6D).

### The effects of microbial therapy on WHR

**Figure 7.**
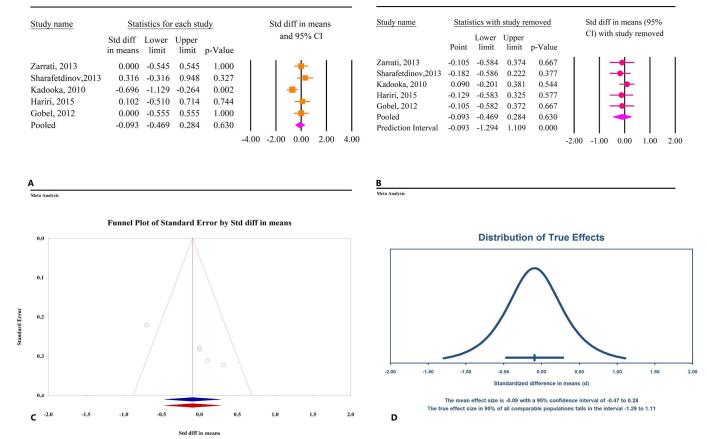
The effects of microbial therapy on WHR in patients with metabolic syndrome. A: Forest plot illustrating the overall effect of microbial therapy on BMI. B: Sensitivity analysis showing the impact of removing individual studies on the overall effect. C: Assessment of publication bias. D: Prediction interval, displaying the expected range of true effects based on the meta-analysis.

The effects of microbial therapy on WHR were not significant (SMD: −0.09; 95% CI: −0.46, 0.28, *P* = 0.63, *I*^2^ = 57.59%) (Fig. 7A). All studies included in this analysis assessed probiotics, with insufficient data available for prebiotics and synbiotics. Sensitivity analysis confirmed that the overall effect remained consistent even after the exclusion of individual studies (Fig. 7B). Egger’s regression test indicated significant publication bias (*P* = 0.003), while the Begg test did not suggest significant bias (*P* = 0.08). The trim-and-fill analysis, conducted without inserting additional studies, produced an SMD of −0.09 (95% CI: −0.46, 0.28) (Fig. 7C). The prediction interval analysis revealed a wide range of true effects, with intervals spanning from −1.29 to 1.11 (Fig. 7D).

### The effects of microbial therapy on weight

**Figure 8.**
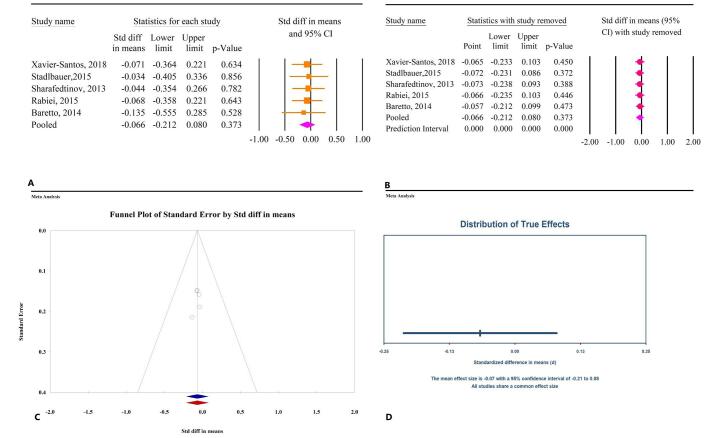
The effects of microbial therapy on weight in patients with metabolic syndrome. A: Forest plot illustrating the overall effect of microbial therapy on BMI. B: Sensitivity analysis showing the impact of removing individual studies on the overall effect. C: Assessment of publication bias. D: Prediction interval, displaying the expected range of true effects based on the meta-analysis.

The meta-analysis findings indicated that microbial therapy did not significantly affect weight in patients with MetS (SMD: −0.06; 95% CI: −0.21, 0.08, *P* = 0.37, *I*^2^ = 0.00%) (Fig. 8A). Similar results were observed with probiotics (SMD: −0.06; 95% CI: −0.27, 0.14, *P* = 0.55, *I*^2^ = 0.00%) and synbiotics (SMD: −0.07; 95% CI: −0.27, 0.13, *P* = 0.50, *I*^2^ = 0.00%), while data on prebiotics were insufficient. Sensitivity analysis confirmed that the overall effect remained consistent even after the exclusion of individual studies (Fig. 8B). Publication bias tests, including Egger’s regression test (*P* = 0.458) and Begg’s (*P* = 1.00), did not reveal significant bias. The trim-and-fill analysis, conducted without the insertion of additional studies, yielded an SMD of −0.06 (95% CI: −0.21, 0.08) (Fig. 8C). The analysis suggests a common ES across all studies, with no significant variation in true effects (Fig. 8D).

### The effects of microbial therapy on HC

**Figure 9.**
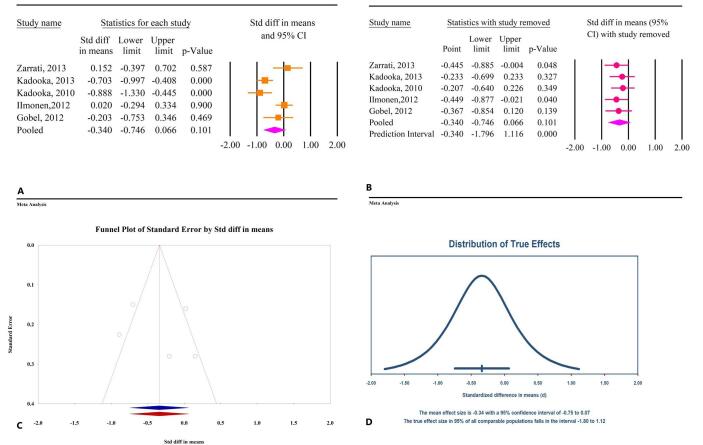
The effects of microbial therapy on HC in patients with metabolic syndrome. A: Forest plot illustrating the overall effect of microbial therapy on BMI. B: Sensitivity analysis showing the impact of removing individual studies on the overall effect. C: Assessment of publication bias. D: Prediction interval, displaying the expected range of true effects based on the meta-analysis.

The meta-analysis revealed no significant effects of microbial therapy on HC in patients with MetS (SMD: −0.34; 95% CI: −0.74, 0.06, *P* = 0.10, *I*^2^ = 79.90%) (Fig. 9A). All the studies included in this analysis assessed probiotics, with insufficient evidence available for prebiotics and synbiotics. Sensitivity analysis confirmed that the overall effect remained consistent even after the exclusion of individual studies (Fig. 9B). Publication bias tests, including Egger’s regression test (*P* = 0.72) and Begg’s test (*P* = 0.62), did not indicate significant bias. The trim-and-fill analysis, conducted without the inclusion of additional studies, was consistent with the main analysis findings (SMD: −0.34; 95% CI: −0.74, 0.06) (Fig. 9C). The prediction interval analysis suggested a broad dispersion among true effects, with prediction intervals ranging from −1.80 to 1.12 (Fig. 9D).

### Meta regression results

**Figure 10.**
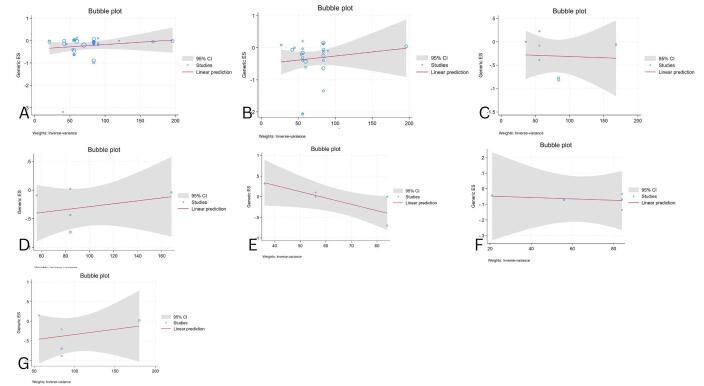
Meta-regression analysis of treatment duration and its association with metabolic syndrome outcomes: A: BMI, B: WC, C: BFM, D: BFP, E: WHR, F: Weight, G: HC. The analysis reveals no significant impact of treatment duration on any of the outcomes.

We performed a meta-regression analysis to evaluate treatment duration’s impact on gut microbial therapy’s effects on MetS-related outcomes. The results showed no significant association between treatment duration and BMI (*P* = 0.35, residual *I*^2^ = 79.95%; Fig. 10A), WC (*P* = 0.47, residual *I*^2^ = 85.16%; Fig. 10B), BFM (*P* = 0.90, residual *I*^2^ = 71%; Fig. 10C), BFP (*P* = 0.56, residual *I*^2^ = 72%; Fig. 10D), WHR (*P* = 0.05, residual *I*^2^ = 28%; Fig. 10E), weight (*P* = 0.88, residual *I*^2^ = 0%; Fig. 10F), or HC (*P* = 0.59, residual *I*^2^ = 80%; Fig. 10G). These findings indicate that treatment duration does not significantly influence these outcomes, despite varying levels of residual heterogeneity.

## Discussion

The rising prevalence of MetS necessitates innovative management strategies. This umbrella review explores the potential of gut microbiome modulation in influencing key anthropometric indices in MetS patients, particularly through the use of probiotics, prebiotics, and synbiotics. By analyzing existing meta-analyses of randomized controlled trials, we highlight the promising results and the persistent limitations in this field. Addressing these limitations could lead to more reliable and conclusive outcomes, paving the way for more effective interventions in MetS management.

Our analysis revealed that microbial therapy significantly reduces BMI and WC in MetS patients. Probiotics, prebiotics, and synbiotics were all associated with a significant reduction in BMI, while only prebiotics and synbiotics demonstrated a significant decrease in WC. There was insufficient evidence to confirm the same effect of probiotics on WC. The impact of microbial therapy on other anthropometric indices, such as BFM, WHR, weight, HC, and BFP, was inconclusive. However, probiotics might potentially lower BFM, HC, WHR, and BFP. At the same time, prebiotics and synbiotics lacked sufficient evidence to confirm a similar effect on these indices in MetS patients.

MetS is characterized by a cluster of conditions, including insulin resistance, central obesity, hypertension, and dyslipidemia^[50,51]^. Various criteria, such as those proposed by the National Cholesterol Education Program (NCEP) Adult Treatment Panel III (ATP III)^[52]^ and the International Diabetes Foundation (IDF)^[53]^ have been used to recognize MetS^[53]^. Researchers have identified several mechanisms that link gut microbiome composition to the development of MetS. It is well established that different diets can influence the gut microbiome, contributing to the onset of MetS. As a result, targeting the gut microbiome is being explored as a potential therapeutic strategy for managing this condition. By altering the gut microbiome, it may be possible to mitigate some of the key factors contributing to MetS, offering a new avenue for treatment^[51,54,55]^.

Diets high in fructose have been linked to an increased risk of diabetes mellitus, cardiovascular disorders, and MetS^[56,57]^. Excess fructose is converted into triglycerides via de novo lipogenesis, leading to elevated lipid levels in the bloodstream and liver. Over time, inflammation and oxidative stress contribute to insulin resistance^[58]^. In a study investigating the dose-dependent effects of specific probiotic strains on MetS induced by a high-fructose diet, a combination of *L. plantarum* KY1032 and *L. curvatus* HY7601 was found to suppress MetS symptoms, though it did not affect body weight^[59]^.

Numerous studies have demonstrated positive health-related outcomes following probiotics, prebiotics, and synbiotics intake. For example, Kadooka *et al* evaluated the effects of the probiotic *Lactobacillus gasseri* SBT2055 (LG2055) on abdominal fat and body weight in adults with obesity tendencies. In a 12-week, randomized, placebo-controlled trial, participants consuming fermented milk with LG2055 experienced significant reductions in abdominal fat, body weight, BMI, waist, and hip measurements compared to a control group. The study concluded that LG2055 has a beneficial impact on reducing abdominal fat and improving metabolic health. Another study further supports this finding, which demonstrated that even low doses of LG2055 in fermented milk significantly reduced abdominal fat, BMI, and body measurements in Japanese adults over 12 weeks. However, the benefits diminished after stopping intake, highlighting the importance of continuous consumption to sustain these effects^[60,61]^.

Additionally, Dewulf *et al* conducted a study to explore the impact of inulin-type fructans (ITF prebiotics) on gut microbiota and metabolism in obese women. In these 3 months, double-blinded, placebo-controlled trial, 30 participants received either ITF prebiotics or a placebo. The findings revealed that ITF prebiotics significantly increased beneficial bacteria, such as *Bifidobacterium* and *Faecalibacterium prausnitzii*, while reducing harmful ones, like *Bacteroides intestinalis* and *Bacteroides vulgatus*. These microbiota changes were linked to slight reductions in fat mass and alterations in plasma metabolites. The study suggests that ITF prebiotics can subtly modify gut microbiota, leading to modest yet meaningful metabolic improvements in obese women^[62]^.

Understanding how gut microbial modulation influences anthropometric indices is complex and necessitates further research. For instance, the gut microbiome is crucial in maintaining the integrity of the intestinal barrier, which prevents systemic inflammation. A healthy barrier stops the absorption of harmful substances like lipopolysaccharides, which can cause low-grade inflammation and contribute to MetS^[63,64]^. Probiotic supplementation can help restore gut homeostasis, improve barrier function, and reduce inflammation-related metabolic disorders^[65,66]^.

Additionally, by fermenting carbohydrates in the gut, probiotics increase the production of short-chain fatty acids (SCFAs) such as acetate, propionate, and butyrate^[67]^. SCFAs, particularly butyrate, nourish intestinal cells, supporting regeneration and strengthening the intestinal mucosa’s protective effects^[68-70]^. SCFAs also interact with G protein-coupled receptors, leading to the secretion of hormones like glucagon-like peptide-1 (GLP-1), peptide YY (PYY), and 5-hydroxytryptamine (5-HT). These hormones help regulate energy absorption, inhibit glucagon release, stimulate insulin secretion, maintain gut barrier integrity, reduce permeability, and control appetite and food intake^[71-74]^.

Moreover, Cani *et al* demonstrated that prebiotic intake can significantly enhance gut health by promoting the growth of beneficial bacteria and influencing gut peptides such as GLP-1 and PYY. These microbiota changes are linked to reduced hunger, improved satiety, better post-meal glucose control, and decreased body fat. These findings suggest that prebiotics also play a vital role in weight management and improving metabolic health through targeted alterations in gut microbiota and hormonal responses.^[75,76]^

The combination of probiotics and prebiotics, known as symbiotics^[77]^, has shown potential in various studies. In a Chinese study, a synbiotic delivered via a dual-core microcapsule (containing a prebiotic shell and probiotics *Lactobacillus* and *Bacillus subtilis*) demonstrated effectiveness in enhancing the gut barrier, reducing liver fat storage and lowering gut inflammation, suggesting its potential as a treatment for MetS induced by a high-fat diet^[78]^. While results on synbiotic effects are sometimes conflicting, many studies have shown their benefits in reducing anthropometric indices such as BMI, WC, visceral fat, and HC in overweight and obese individuals^[77]^.

For example, the study by Safavi *et al* investigated the effects of synbiotic supplementation on obesity and metabolic markers in children and adolescents. The results showed that synbiotic intake significantly reduced the participants’ BMI z-scores, WC, and WHR. These findings suggest synbiotics could be a promising adjunctive treatment for obesity in younger populations.^[79]^. Similarly, Sanchez *et al* explored the effects of *Lactobacillus rhamnosus* CGMCC1.3724 (LPR) on weight loss and maintenance in obese men and women over 24 weeks. Participants consumed a synbiotic formulation containing LPR (a probiotic), oligofructose, and inulin (prebiotics). While overall weight loss was similar across groups, women in the LPR group experienced significantly greater weight and fat loss than those in the placebo group, especially during the maintenance phase. LPR was also linked to reductions in fat mass, leptin levels, and increased beneficial gut bacteria, suggesting its potential for sustainable weight loss in women^[80]^.

Isolating the role of gut microbiota in MetS is particularly challenging due to the intertwined effects of genetics, diet, lifestyle, antibiotic use, and the diverse composition of gut microbiomes among individuals^[81-83]^. This complex relationship between gut microbiota and obesity has led to inconsistent study results. To address these inconsistencies, Magne *et al* highlighted the need for more detailed participant characterization to better identify influencing co-variables. The notion of a single microbial signature for obesity is becoming increasingly uncertain. Future research should instead focus on identifying microbial patterns that can categorize patients into subgroups, enabling more personalized and effective obesity treatments through targeted microbiome interventions^[84]^.

Assessing heterogeneity and publication bias is essential for accurately interpreting the role of microbial therapy in modifying anthropometric indices. No publication bias was detected except in the WHR subgroup analysis, as indicated by Egger’s test. Significant heterogeneity was observed across studies for most anthropometric indices, except for weight. This variability can be attributed to several factors, including differences in study populations, intervention protocols, dietary and lifestyle factors, methodological approaches, baseline gut microbiota composition, and participant adherence. Variations in age, sex, ethnicity, and health status among participants can lead to differing responses to microbial interventions. For instance, gut microbiota composition and MetS components have been shown to vary across ethnicities, potentially affecting treatment outcomes. Additionally, the diversity in the type of microbial therapy, dosage, duration, and delivery methods across studies can result in inconsistent effects on metabolic parameters. Dietary habits and physical activity levels further modulate gut microbiota composition and function, interacting with microbial therapies and influencing their efficacy. Methodological differences, such as study design, sample size, and outcome measurement techniques, introduce further heterogeneity. Moreover, the baseline composition of participants’ gut microbiota can affect their response to interventions, as individuals with different gut microbial profiles may exhibit varied metabolic responses to the same treatment. Finally, varying levels of adherence to intervention protocols among participants can impact outcomes and contribute to heterogeneity. Addressing these sources of variability is crucial for accurately interpreting the efficacy of gut microbial therapies for MetS. Future research should aim to standardize intervention protocols, control for confounding factors, and consider personalized approaches based on individual microbiota profiles to reduce variability and enhance the reliability of findings.

The studies reviewed exhibited varying levels of quality. For instance, Arabi *et al*^[48]^ found that only a third of the included studies had a “good” overall quality, with significant BMI reductions observed mainly in the higher-quality studies. The definition of MetS varied across meta-analyses, with different criteria (NCEP ATP-III, IDF) or not being reported at all. Study durations ranged from a single administration to 28 weeks, and the types of microbial therapy varied widely, including food, fecal microbial transplantation, capsules, and more. Some studies also incorporated additional interventions, such as physical activity or specific diets^[36]^.

While most studies included male and female participants from various age groups, a few had single-gender control groups. Some, like Dong *et al*^[34]^, included at-risk postpartum and pregnant women. The gut microbiome changes during different pregnancy trimesters to support fetal development, with overweight and obese pregnant women typically showing increases in *Firmicutes* and *Bacteroidetes*^[85]^. It is also important to note that some of the included studies were from both Western and Asian societies, so race and ethnicity, geographical conditions, and different cultures affecting daily diets must also be considered to accurately interpret the results^[36]^. Beyond the influence of differing diets, which account for 57% of an individual’s gut microbiome, genetics also play a significant role, contributing 27% to gut microbiome composition. This genetic influence can further impact the outcomes of microbial interventions in study participants^[86]^.

The present review consolidates evidence from multiple meta-analyses, offering a comprehensive evaluation of microbial therapy’s role in modifying anthropometric indices among MetS patients. While previous studies have explored this relationship, they often exhibit methodological heterogeneity, including differences in probiotic strains, dosages, intervention durations, and study populations. Furthermore, the interaction between gut microbiota composition and MetS components remains an area of ongoing investigation. By synthesizing these findings, this review highlights the need for standardized intervention protocols, rigorous methodological frameworks, and further research into personalized microbial therapy based on individual gut microbiota profiles. Addressing these gaps will enhance the applicability of microbial interventions in clinical settings.

## Strengths and limitations

This umbrella review provides valuable insights into the potential effects of gut microbiome modulation on anthropometric indices in individuals with MetS. A key strength of this study lies in its systematic approach, synthesizing evidence from multiple meta-analyses to evaluate the effects of probiotics, prebiotics, and synbiotics. Subgroup analyses allowed us to explore the distinct effects of these interventions and investigate the underlying mechanisms influencing outcomes. Our findings suggest that gut microbiome modulation positively impacts BMI and WC, which are critical indicators in MetS management. These findings underline the potential of microbial therapies as adjunctive treatments for MetS.

Despite these strengths, the study has several limitations. First, the number of included meta-analyses was relatively small, restricting the scope of synthesized evidence and preventing subgroup analyses based on study quality. This limitation was particularly notable given that some included studies were rated as critically low quality. Although sensitivity and publication bias analyses offered valuable insights, a larger number of high-quality meta-analyses are required for more robust conclusions.

Additionally, significant heterogeneity was observed across the included studies for most anthropometric indices, except for weight. To explore this heterogeneity, we conducted a meta-regression analysis to evaluate the impact of treatment duration on outcomes, but no significant associations were found except for WHR. A meta-regression analysis based on treatment doses was also considered, but inconsistencies in reporting prevented its implementation. Doses were reported in varying units, such as milligrams (mg) or colony-forming units (CFU). Furthermore, the type of intervention varied widely, including pills, milk-based products, and other delivery methods. These inconsistencies highlight the need for standardized reporting of doses, intervention types, and protocols in future research.

Other limitations include variability in study designs, populations, and baseline gut microbiota compositions. Differences in intervention durations, participant demographics, and quality assessment methodologies added complexity to the synthesis and interpretation of results. The lack of detailed participant characterization, such as dietary habits, genetic factors, and baseline microbiota profiles, limited our ability to explore the personalized effects of microbial therapies. These factors, combined with the multifactorial nature of MetS, underscore the need for more standardized methodologies, including consistent microbial therapy doses and strains, study durations, and consideration of demographic and microbiome composition.

While the current evidence is promising, implementing these interventions in clinical practice may require complex strategies with careful monitoring. There is no definitive agreement on targeted therapies for adjusting the gut microbiome in individuals with MetS, highlighting the need to address these limitations in future research. By enhancing participant characterization and addressing the variability in existing studies, future research can better explore the potential of microbial therapy in managing MetS and obesity.

## Conclusion

In this meta-umbrella study, we highlighted the promising effects of gut microbial modulation on BMI and WC in patients with MetS by administering probiotics, prebiotics, and synbiotics. In contrast, these compounds did not significantly affect other anthropometric indices. Given that obesity is a key risk factor in the development of MetS, gut microbial modulation may serve as a valuable adjunctive therapy alongside lifestyle modifications for managing MetS. However, further high-quality studies with standard methodologies and longer follow-up duration are required to reach more conclusive findings regarding the effects of gut microbial modulation in patients with MetS.

## Data Availability

The datasets used and/or analyzed during the current study are accessible from the corresponding author on reasonable request.

## Research registration unique identifying number (UIN)

The study was registered in PROSPERO with the registration code CRD42022346998.
